# Importance of metabolic rate to the relationship between the number of genes in a functional category and body size in Peto's paradox for cancer

**DOI:** 10.1098/rsos.160267

**Published:** 2016-09-07

**Authors:** Kazuhiro Takemoto, Masato Ii, Satoshi S. Nishizuka

**Affiliations:** 1 Department of Bioscience and Bioinformatics, Kyushu Institute of Technology, Iizuka, Fukuoka 820-8502, Japan; 2 Molecular Therapeutics Laboratory, Department of Surgery, Iwate Medical University School of Medicine, Morioka, Iwate 020-8505, Japan

**Keywords:** cancer, Peto's paradox, functional analysis of genome, metabolic rate, body size

## Abstract

Elucidation of tumour suppression mechanisms is a major challenge in cancer biology. Therefore, Peto's paradox, or low cancer incidence in large animals, has attracted focus. According to the gene-abundance hypothesis, which considers the increase/decrease in cancer-related genes with body size, researchers evaluated the associations between gene abundance and body size. However, previous studies only focused on a few specific gene functions and have ignored the alternative hypothesis (metabolic rate hypothesis): in this hypothesis, the cellular metabolic rate and subsequent oxidative stress decreases with increasing body size. In this study, we have elected to explore the gene-abundance hypothesis taking into account the metabolic rate hypothesis. Thus, we comprehensively investigated the correlation between the number of genes in various functional categories and body size while at the same time correcting for the mass-specific metabolic rate (*B*_c_). A number of gene functions that correlated with body size were initially identified, but they were found to be artefactual due to the decrease in *B*_c_ with increasing body size. By contrast, immune system-related genes were found to increase with increasing body size when the correlation included this correction for *B*_c_. These findings support the gene-abundance hypothesis and emphasize the importance of also taking into account the metabolic rate when evaluating gene abundance–body size relationships. This finding may be useful for understanding cancer evolution and tumour suppression mechanisms as well as for determining cancer-related genes and functions.

## 1. Introduction

Cancer is a complex and robust disease that remains difficult-to-treat despite the development of numerous anti-cancer therapies [1,2]. Elucidation of cellular tumour suppression mechanisms is a long-standing goal for cancer researchers. In recent years, Peto's paradox, first reported in 1975 [3], has attracted renewed attention to this topic [4]. This paradox is often explained as follows: assuming that the probability of normal cells transitioning to cancer cells is equivalent among all cells, larger animals (i.e. organisms with more cells) are expected to show a higher incidence of cancer. However, a positive correlation between body size (or mass) and cancer incidence has not been observed [4–6]. In fact, previous studies have reported no association between body size and cancer incidence (and also the lifespan-adjusted equivalent) [7,8]. These results indicate the existence of body-size-dependent mechanisms of tumour suppression.

Several hypotheses have been proposed to resolve Peto's paradox (or to explain body-size-dependent suppression mechanisms). The metabolic rate hypothesis [9,10] is particularly interesting because cancer cells have altered glucose metabolism in that they typically possess enhanced aerobic glycolysis (the so-called Warburg effect). This hypothesis states that larger animals have relatively lower cancer incidence because the metabolic (oxygen consumption) rate per unit body mass, which is roughly equal to the rate at the cellular level, decreases with body size. Oxygen radicals often enhance ontogenetic mutations and induce the transition from normal cells to tumour cells. In short, large animals have lower cancer rates, because they can avoid oxidative stress and subsequent genetic mutations. In fact, oxidative DNA damage is associated with metabolic rate [11], and the mutation rate decreases with body size because of the decrease in the mass-specific metabolic rate [12,13]. The negative correlation between mass-specific metabolic rate (*B*_c_) and body mass (*M*) is well known in the context of Kleiber's Law (or the allometric scaling of metabolic rate) [14–16]; in particular, the relationship between metabolic rate (*B*) and body mass is generally described as a power-law function: *B* ∝ *M*^3/4^. Thus, mass-specific metabolic rate shows a power-law decay with body mass: *B*_c_ = *B*/*M* ∝ *M*^−1/4^. The metabolic rate hypothesis may show promise for resolving Peto's paradox; however, it is unsuitable for investigating the cellular (microscopic) mechanisms of tumour suppression, because it is based on physiology (macroscopic behaviour). In addition to this, the basis for allometric scaling of metabolic rate is still unclear even though several hypotheses, or models, have been proposed [17–21].

Genetic studies are useful in this context. In particular, a number of cancer-related genes (e.g. tumour suppressor genes (TSGs) and oncogenes) and pathways [22,23] have been identified by several new technologies including the use of high-throughput methods. Previous studies have proposed an alternative hypothesis: the *gene-abundance hypothesis* [5,24,25], which states that larger animals have more (and/or more highly expressed) genes that suppress cancer progression (e.g. TSGs) and fewer (and/or more lowly expressed) genes that induce cancer growth. Further theoretical and experimental studies have provided deeper insights into this hypothesis. For example, a theoretical study [24] showed that the number and/or expression levels of (proto-)oncogenes decreases with increasing body size, whereas the number and/or expression levels of TSGs are hardly correlated with body size because of energetic limitations. In particular, the increase of TSGs requires energetic costs resulting from repairing mutations and reproduction; thus, such an evolutionary strategy (i.e. the increase of TSGs) for cancer suppression is hardly adopted (see [24] for details). A comparative genomic study [26] demonstrated that bowhead whale-specific gene mutations were linked to cancer and ageing; moreover, it reported that duplications frequently occurred in genes related to DNA-repair, cell cycle and ageing. Another study [8] showed that elephants have a larger TP53 copy number, which is a well-known TSG, compared with other mammals, and also reported that elephant cells are more resistant to DNA damage, than human cells. Retroviral integration can also induce cancer. A previous study [27], based on bioinformatics and mathematical biology, has shown that the levels and activity of endogenous retroviruses (ERVs) acquired in the last 10 Myr decrease with body size; it was also reported that the mean age of ERVs increases with body size. Inspired by the fact that cancer cell mutations directly increase the number of microsatellites in tumour DNA [28], researchers have revealed that the number of microsatellites decreases with body size [29]. Taken together, these results support the gene-abundance hypothesis.

However, a more careful examination may be required to prove the validity of the gene-abundance hypothesis. Previous studies have only focused on the relationship between specific biological functions and body size, and they generally compared functions among a few mammals. The effect of metabolic rate (i.e. the metabolic rate hypothesis) also needs to be considered when investigating the gene abundance–body size relationships. The gene-abundance hypothesis may therefore be overlapping with the metabolic rate hypothesis. In particular, it remains possible that the gene abundance–body size relationships are spurious correlations that result from the body-size-dependent metabolic rate. For example, the number of genes in a functional category (NOGF) generally shows a positive correlation with the mass-specific metabolic rate [30] (i.e. a number of functional categories are associated with mass-specific metabolic rate). This fact predicts that NOGF will generally show a negative correlation with body size because of the negative association between mass-specific metabolic rate and body size. Moreover, oncogenes and tumour suppressors are known to regulate metabolism (reviewed in [9,10]). For example, Myc is an oncogene that is known to regulate glycolysis and to activate mitochondrial biogenesis, whereas the p53 protein, which is a tumour suppressor, is known to inhibit glycolysis. Glycolysis and biogenesis are strongly related to respiration (i.e. metabolic rate). Assuming that oncogenes generally increase the metabolic rate at the cellular level and that TSGs decrease it, the number of oncogenes and TSGs are expected to show negative and positive associations with body size, respectively. This expectation is consistent with the prediction from the gene-abundance hypothesis.

Therefore, we performed an integrated evaluation of the metabolic rate hypothesis and the gene-abundance hypothesis. In particular, genomic data, metabolic rate and body size for mammals were collected from public databases and the literature, and using statistical methods, the relationships between NOGF and body size were investigated by removing the effect of mass-specific metabolic rate.

## 2. Material and methods

### 2.1. Data on metabolic rate, body mass and genome

We used data on mammalian metabolic rates and body masses obtained in our previous study [30]. Additionally, we also collected data for those mammals whose genomes were available in a species-level database within the Kyoto Encyclopedia of Genes and Genomes (KEGG) database [31]. The units of mass-specific metabolic rate and body mass were converted to watts per gram (W/g) and grams (g), respectively. To reduce the phylogenetic signals, one representative species was selected from each genus, according to the year in which the species genome sequence was first completed. Finally, we obtained data for 33 mammals (electronic supplementary material, table S1).

### 2.2. Functional categories of genes

The method we used is almost similar to that reported in our previous study [30]. We used the third level of KEGG BRITE Functional Hierarchy [32] in the KEGG metabolic map (www.genome.jp/kegg-bin/get_htext?br08901.keg) for measuring the number of genes in functional categories. We downloaded the data on functional category–gene identifier relationships of species S from the KEGG FTP site (ftp.bioinformatics.jp/kegg/brite/organisms/S/S00001.keg) on 9 May 2015, where S corresponds to the KEGG organism identifier (electronic supplementary material, table S1). In this study, we did not consider Gene Ontology (GO) [33] as an alternative definition of functional categories because there were fewer organisms whose annotations in the GO database were completed, compared to those in the KEGG database. In addition, we selected TSGs and oncogenes identified in the literature [22,23]. In total, we investigated 342 functional categories.

### 2.3. Statistical analysis

Statistical analysis was performed using R software (v. 3.2.4; www.r-project.org). The association between NOGF and body size/mass-specific rate was evaluated based on the Pearson's product-moment correlation coefficient *r* and its associated *p*-value. Mass-specific metabolic rate and body mass were log-transformed for all analyses. To evaluate NOGF–body size correlations when the mass-specific metabolic rate was kept constant, the partial correlation analysis was considered; in particular, we used the *pcor* function that is available in the R package *ppcor* (v. 1.1). Additionally, we also considered a multivariate analysis; in particular, the standardized partial regression coefficient for *M* in the formula NOGF ∼ *M* + *B*_c_ (i.e. the *B*_c_-corrected estimate) was calculated for each functional category using the *lm* function (electronic supplementary material, tables S2 and S3). We used the *lm* function to calculate the residuals for variables.

To find the functional categories that are specifically associated with body size, we used an enrichment analysis based on Fisher's exact test, inspired by gene set enrichment analysis [34]. In particular, for the KEGG BRITE Functional Hierarchy we focused on the second and third functional hierarchical levels and calculated the statistical significance, defined as −log_10_(*p*-value using Fisher's exact test), for the ratio of functional categories (i.e. the categories at the third level) correlated with body size (*p* < 0.05) to all the functional categories in the upper (i.e. second-level) category. The *fisher.test* function was used to perform Fisher's exact test. The changes in the *p*-value threshold for the NOGF–body size correlations did not significantly affect the conclusion.

### 2.4. Phylogenetically independent contrasts

Phylogenetically independent contrasts (PICs) [35] of the variables were evaluated in order to remove any possible phylogenetic effect on the association between biological variables in the context of the phylogenetic comparative analysis [36,37]. This method is similar to that used in our previous study [38]. In particular, the mammalian phylogenetic tree was constructed using the matrix extracellular phosphoglycoprotein precursor (MEPE) gene (electronic supplementary material, figure S1), downloaded from the KEGG database on 20 August 2015. Note that the platypus, *Ornithorhynchus anatinus*, was omitted from the tree because the MEPE gene for this species was not found. Based on the phylogenetic tree, the PICs were calculated using the function *pic* in the R package *ape* (v. 3.4).

## 3. Results

### 3.1. The number of genes in functional categories generally show a negative correlation with body size

We found that NOGF was generally associated with body size. Genome size and the total number of genes (proteins) did not affect the observed NOGF–body size correlations because body size did not correlate with either genome size (*r* *=* −0.054, *p* = 0.77) or number of proteins (*r* = −0.17, *p* = 0.35).

**Figure 1.**
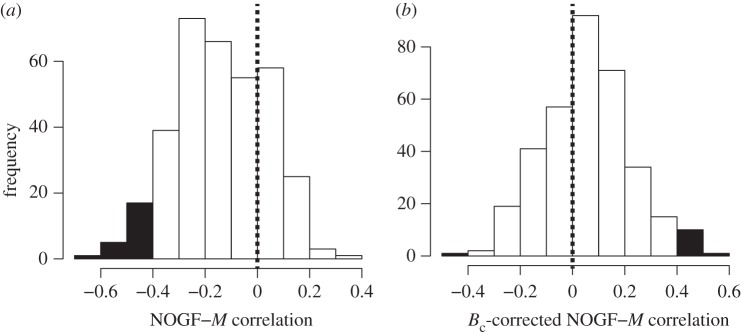
Distribution of the correlation coefficient between the NOGF and body mass (*M*). Mass-specific metabolic rate (*B*_c_) correction was (*a*) not considered (*b*) considered. The filled area indicates the *p*-value of less than 0.05.

In general, the associations observed were negative (figure 1*a*). In particular, the mean of *r* was −0.14 (95% CI, from −0.16 to −0.12). About 13% (43/342) of the functional categories showed a statistically significant correlation with body size (*p* < 0.05; also the electronic supplementary material, table S2). For example, the number of oncogenes decreases with body size (*r* = −0.38, *p* = 0.031); by contrast, the number of TSGs shows no correlation with body size (*r* = −0.28, *p* = 0.11). This result supports the gene-abundance hypothesis and is consistent with the theoretical prediction [24] that the number of oncogenes decreases with body size, whereas the number of TSGs is almost independent of body size due to reproduction. In addition, disease incidence (and by inference disease-related genes) also decreases with increasing body size. For instance, body size was negatively associated with viral myocarditis (*r* = −0.63, *p* = 8.0 × 10^−5^), type I diabetes mellitus (*r* = −0.57, *p* = 6.1 × 10^−4^), and graft-versus-host disease (*r* = −0.55, *p* = 3.7 × 10^−3^). These observed negative associations could be considered reasonable because these diseases possibly arise due to cancer treatment and/or because they increase the incidence of cancer [39–41]. Therefore, this result also supports the gene-abundance hypothesis.

### 3.2. Body-size-dependent metabolic rate strongly influences correlations between the number of genes in functional categories and body size

A conclusion that the gene-abundance hypothesis is correct needs to be made with caution, because it remains possible that the observed associations are spurious. For some of the functional categories, the NOGF–body size relationships were found to be questionable. For example, the relationship between cancer and transduction of taste signals could not be explained biologically although a negative correlation between NOGF and body size was clearly observed (*r* = −0.57, *p* = 6.9 × 10^−4^; see also §3.3). Similarly, the association between serotonergic synapses and body size (*r* = −0.48, *p* = 5.2 × 10^−3^) could also not be readily explained in the context of tumour biology. We can, however, speculate that the observed association may suggest a role for serotonin in cancer, because low doses of serotonin are known to inhibit cancer growth by decreasing tumour blood supply [42]. It should be noted that blood supply is positively correlated with metabolic rate [15,17]. In summary, these observations suggest the possibility that genetic changes may have an indirect effect on cancer. In particular, the genetic change does not contribute directly to the inhibition or enhancement of cancer growth; rather it implies that genetic changes (e.g. increase/decrease in NOGFs) lead to a change in metabolic rate, which then influences cancer growth. Therefore, the noted differences in metabolic rate according to body size should be taken into consideration when evaluating NOGF–body size associations. In particular, NOGFs are expected to show negative correlations with body size, because they are positively correlated with mass-specific metabolic rate which itself is negatively correlated with body size. In fact, all the statistically significant correlations that were observed, were negative (figure 1*a*); moreover, the observed NOGF–body mass negative correlation was not concluded when corrected with the mass-specific metabolic rate using the partial correlation analysis (figure 1*b*).

**Figure 2.**
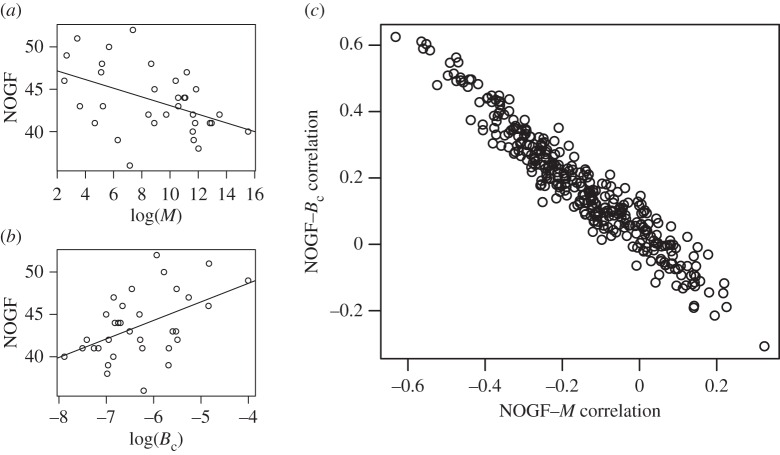
Relationship between the NOGF, body mass (*M*) and mass-specific metabolic rate (*B*_c_). Correlations of NOGF for fatty acid degradation with (*a*) log(*M*) and (*b*) log(*B*_c_). (*c*) Scatter plot of the correlation coefficient (*r*) between NOGF and *M* versus *r* between NOGF and *B*_c_.

Additional results suggest the influence of metabolism on the NOGF–body size relationship. In particular, functional categories related to metabolism were frequently observed (see the following section for details). For example, body size was linked to lipid metabolism (e.g. fatty acid degradation (figure 2*a*; *r* = −0.46, *p* = 6.7 × 10^−3^) and fatty acid biosynthesis (*r* = −0.50, *p* = 3.3 × 10^−3^)) as well as nucleotide (pyrimidine) metabolism (*r* = −0.52, *p* = 1.7 × 10^−3^). Lipid metabolism [10,43] and nucleotide metabolism [10,44] are known to play important roles in cancer. However, their role in cancer is often considered in the context of energy regulation, which of course is related to metabolic rate [16]. In addition, fatty acids are believed to determine metabolic rate (the so-called *membrane-pacemaker hypothesis* [45–47]). In fact, the number of genes involved in lipid metabolism is known to be correlated with the mass-specific metabolic rate [30]. In our dataset, the number of genes involved in fatty acid degradation was also positively correlated with mass-specific metabolic rate (figure 2*b*; *r* = 0.49, *p* = 3.4 × 10^−3^).

For all functional categories, we calculated the correlation coefficient between the NOGF–body mass (*M*) correlation and the NOGF–mass-specific metabolic rate (*B*_c_) correlation (figure 2*c*). Assuming that the mass-specific metabolic rate influences the NOGF–*M* correlation, a negative association between the NOGF–*M* correlation and the NOGF–*B*_c_ correlation was observed due to the observed negative correlation between mass-specific rate and body mass (*r* = −0.95, *p* < 2.2 × 10^−16^): *B*_c_ ∝ *M*^−0.24^ ^±^ ^0.01^. As expected, this negative association was confirmed (figure 2*c*; *r* = −0.96, *p* < 2.2 × 10^−16^). This result indicates that the observed NOGF–*M* correlations result from the *B*_c_–*M* correlation.

### 3.3. Immune system-related genes increase with body size when the mass-specific metabolic rate is kept constant

**Figure 3.**
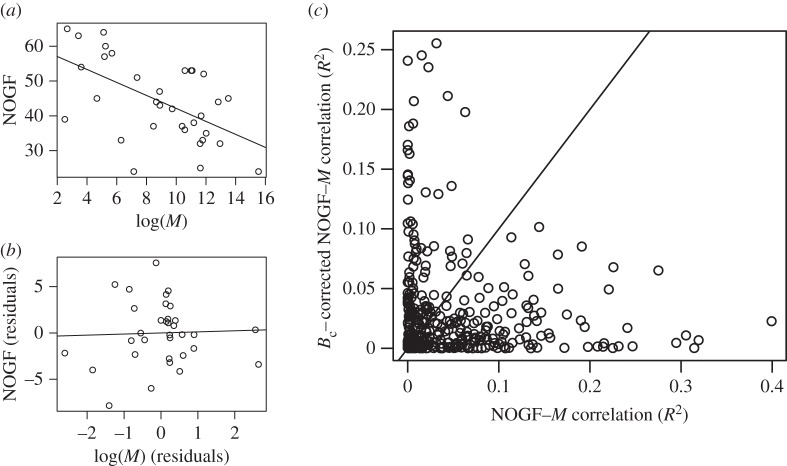
Change in the relationship between the NOGF and body mass (*M*). (*a*) NOGF–*M* correlation for taste transduction. (*b*) NOGF–*M* correlation for taste transduction corrected with the mass-specific metabolic rate (*B*_c_). (*c*) Scatter plot for the determination of coefficient (*R*^2^) of NOGF–*M* correlation versus *R*^2^ of NOGF–*M* correlation corrected with *B*_c_. The solid line indicates the diagonal line.

**Figure 4.**
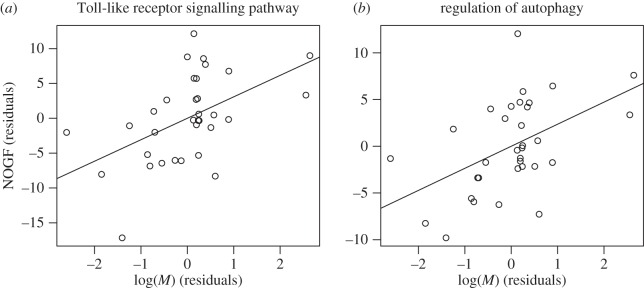
Scatter plot of the NOGF (residuals) versus log-transformed body mass (*M*) (residuals). (*a*) Toll-like receptor signalling pathway. (*b*) Regulation of autophagy.

We used a partial correlation analysis to eliminate the effect of the mass-specific metabolic rate on the NOGF–*M* correlations, and found that the NOGF–*M* associations for functional categories changed significantly (figure 3). In particular, the NOGF–*M* associations, identified by a simple correlation analysis, were not observed when the effect of metabolic rate was considered. For example, the observed NOGF–*M* correlation for taste transduction (figure 3*a*; *r* = −0.57, *p* = 6.9 × 10^−4^) was not observed when corrected for the mass-specific metabolic rate (figure 3*b*; *r*_p_ = 0.013, *p* = 0.94). This indicates that most of the observed correlations were artefacts likely occurring due to the body-size-dependent mass-specific metabolic rate. Moreover, the mean of *r*_p_ was positive (0.054 (95% CI, from 0.036 to 0.072); see also figure 1*b*), unlike in the case of simple correlation analysis where the metabolic rate effect was not considered (figure 1*a*). Around 6% (20/342) of the functional categories showed a statistically significant correlation with body size (*p* < 0.05; see also the electronic supplementary material, table S2). Representative examples of the functional categories that were correlated with body mass when the mass-specific metabolic rate was kept constant are shown in table 1. For example, the increase in the number of genes in the Toll-like receptor signalling pathway (figure 4*a*) may contribute to cancer resistance, because this pathway plays an important role in host defence from infection [48]. The other signalling pathways, identified based on NOGF–body size correlations, are also expected to contribute to the resistance against cancer. In particular, the IgE-mediated allergic reaction mediated by FcεRI signalling has been proposed as a target for cancer immunotherapy [49]. The RIG-I-like receptor signalling pathway has been shown to be activated in cancer therapies through endogenous non-coding RNAs [50]. The increase in the number of genes involved in the regulation of autophagy (figure 4*b*) may be explained in the context of the protective function of autophagy to limit tumour necrosis and inflammation [51]. Since natural killer cells contribute to the killing of tumour cells without a required immunization or deliberate activation [52], the increase in the number of natural killer cell genes that mediate cytotoxicity can be expected to enhance resistance against cancer. Cytokine interactions are also known to be important in the context of cancer pathogenesis and cancer therapy [53]. It should be noted that these functional categories were identified only when the correlation based on mass-specific metabolic rate was considered.

**Table 1.** Correlations between the NOGF and body mass. The partial (mass-specific metabolic rate-corrected) correlation coefficients (*r*_p_) and correlation coefficients (*r*) are shown. The functional categories are only represented when the *p*-value is less than 0.01 using the partial correlation analysis (see electronic supplementary material, table S2 for the full version), and they are displayed in descending order of *r*_p_. Parenthetic values indicate the *p*-values.

upper category	functional category	*B*_c_-corrected *G*–*M* correlation *r*_p_ (*p*-value)	*G*–*M* correlation *r* (*p*-value)
immune system	toll-like receptor signalling pathway	0.51 (3.2 × 10^−3^)	0.18 (0.32)
immune system	FcεRI signalling pathway	0.50 (4.0 × 10^−3^)	0.12 (0.49)
transport and catabolism	regulation of autophagy	0.49 (4.4 × 10^−3^)	0.0029 (0.99)
immune system	RIG-I-like receptor signalling pathway	0.49 (4.9 × 10^−3^)	0.15 (0.40)
immune system	natural killer cell-mediated cytotoxicity	0.46 (8.1 × 10^−3^)	−0.21 (0.24)
signalling molecules and interaction	cytokine–cytokine receptor interaction	0.45 (9.0 × 10^−3^)	0.082 (0.65)

**Figure 5.**
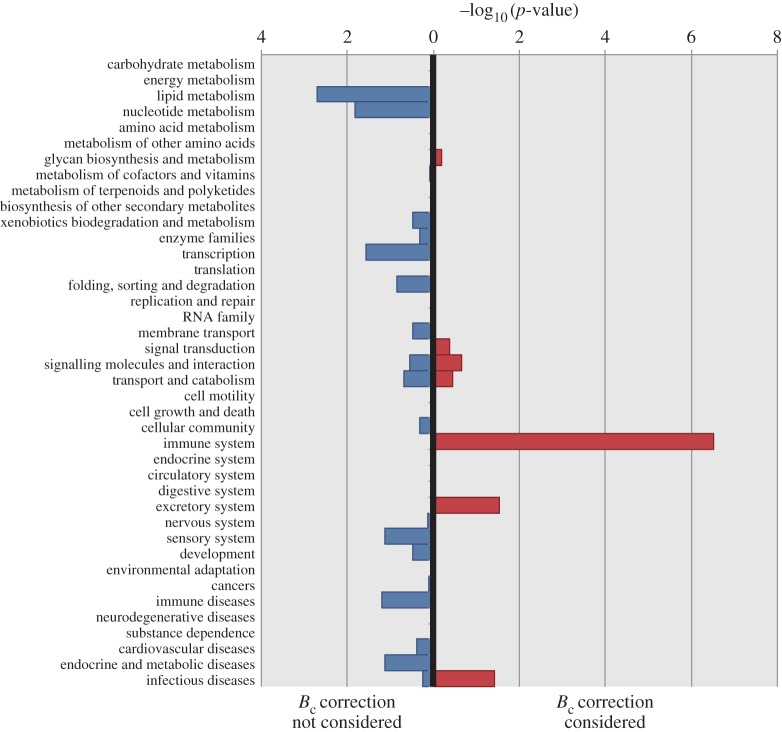
Enrichment of functional categories correlated with body size. The blue bars and red bars indicate the case that mass-specific metabolic rate (*B*_c_) correction was not considered for evaluating the relationships between the NOGF and body mass (*M*) and the case that *B*_c_ correction was considered for evaluating NOGF–*M* correlations, respectively.

The functional categories that were correlated with body mass were enriched in the immune system (red bars in figure 5). This result indicates that the number of immune system-related genes generally increases with body size. On the contrary, no clear pattern was found when the effect of metabolic rate was ignored (blue bars in figure 5) even though such functional categories were enriched in lipid and nucleotide metabolism.

We did not consider PICs in the above analyses because of the alternative methodology we used (see §2.4 for details) and because there are limitations to phylogenetic comparative analysis. In particular, phylogenetic comparative analysis assumes a Brownian motion-like evolution of biological traits on a phylogenetic tree with accurate branch lengths and may thus result in misleading conclusions. In addition, statistical power decreases when a dataset is reduced in size following phylogenetic corrections [54]. Despite these caveats, almost identical conclusions were obtained when PICs were considered (electronic supplementary material, table S3). Therefore, phylogenetic effects did not influence the outcome of this study.

## 4. Discussion

We comprehensively evaluated the gene-abundance hypothesis in the context of the metabolic rate hypothesis, and found that NOGF generally showed a negative correlation with body size. However, such associations were found to be artefactual due to the relationship between metabolic rate and body mass. In particular, the result of the NOGF–body size correlations was significantly different when the analysis was corrected for metabolic rate. Moreover, NOGF tended to be related to metabolic rate rather than to cancer. When evaluating the NOGF–body size relationships corrected for metabolic rate, we found that immune system-related genes increased with body size. A number of previous studies support the notion that gene functions identified based on metabolic rate-corrected NOGF–body size relationships are reasonable in the context of tumour suppression and cancer therapies. This result was obtained only when the metabolic rate was kept constant, indicating that cancer-related genes/functions could easily be overlooked when the difference in metabolic rate according to body size is not considered. These results emphasize the importance of metabolic rate in evaluating gene abundance–body size relationships.

In recent years, researchers in this area have focused on the genomes of large animal (e.g. whales [26] and elephants [8]) to test the gene-abundance hypothesis. However, such studies did not consider the effect of metabolic rate and therefore may provide misleading conclusions. Our results do not contradict those from previous studies because of the strong relationship between metabolism and cancer [10]; however, the NOGF–body size associations identified when the effect of metabolic rate was ignored should be considered in the context of the metabolic rate hypothesis rather than the gene-abundance hypothesis. Our results provide significant support for the gene-abundance hypothesis. In particular, we have shown that the hypothesis is supported in a broad range of mammals, even though previous studies have generally focused on large animal to small animal comparisons.

However, more careful analyses may be required in further studies. For example, our conclusion (the importance of immune system, in particular) is still debatable because we only performed correlation analyses. In this context, a multivariate analysis would be more appropriate. However, in this study we could not perform such an analysis because of the multi-collinearity that arises from the fact that there was gene overlap among functional categories. In fact, we were unable to calculate partial regression coefficients and associated *p*-values because of singularities in the generalized linear model. As mentioned in our previous study [30], this is a particular problem in GO analysis. To resolve this problem, for example, we will need to define alternative (e.g. non-overlapping) functional categories (see below for details). Despite these limitations, the bulk of the experimental evidence supports our conclusions.

It should also be noted that we evaluated NOGFs–body size associations by assuming that the Peto's paradox is correct. However, ideally, we need to consider the relationship with actual cancer incidence rates in animals. In this study, we did not consider the actual cancer incidence rates because only limited data are available. However, recent studies have begun to investigate cancer rates in wildlife (e.g. reviewed in [55]). The data on cancer risks obtained in these studies will be very useful in further studies. In addition to this, factors like lifespan, generation time and mutation rate are also different across species; however, because it is believed that these biological parameters are related to metabolic rate, a correction based on mass-specific metabolic rate may be the most useful one to remove most of the effects of these biological parameters [11,13,16,56]. A critical review [57] claims that Peto's paradox relies on several questionable assumptions, and it emphasizes the importance of organ-level comparisons rather than species-level comparisons in addressing the variation in cancer risk across tissues [58]. The differences in risk may be explained in terms of differences in the number of tissue cell divisions [58] as well as differences in the robustness of cancer signalling networks [59,60].

Another consideration is that the definition of gene abundance is controversial. For simplicity, we have only considered the NOGF as an indicator of gene abundance. However, gene abundance could also be measured in terms of expression level, copy number and activity of functional genes. Indeed, a previous study reported that elephants show a higher expression level and copy number of TP53, compared to humans. It is also possible that the body-size-dependent metabolic rate can give rise to differences in gene expression and copy number, because the expression levels of (enzymatic) genes are also known to determine metabolic rate [61,62]. Thus, the correction for metabolic rate may also be required when considering the relationships between body size and expression level, copy number and/or activity. We attempted to consider expression levels using gene expression data from various mammalian organs [63]; however, a comprehensive analysis was not possible because of the limited amount of data available. The measurement of gene abundance at the organ level using high-throughput techniques (e.g. microarray and RNA-Seq [64]) should therefore be undertaken for a wide range of organisms in the future.

As is the case for other genome analysis studies, our study has some general limitations. For example, we only considered organisms for which genome sequences were completed and available; thus, our species selection was unintentionally biased. In this context, metagenomic techniques may help to complete the genomes of more organisms. Moreover, our findings depend significantly on the quality of genome annotation. The functional categories, defined by the KEGG BRITE Functional Hierarchy in this study, might be somewhat arbitrary (i.e. they depend on the opinions of database administrators). Computational frameworks may increase the quality of genome annotation in terms of protein function prediction (e.g. machine learning-based methods [65]); they may also provide alternative definitions of functional categories. For example, computational methods based on the following frameworks may be useful; graph clustering or community detection of networks [66], biosynthetic capability [67], gene clusters and chemical transformation patterns [68,69].

Despite the limitations of our data analysis, these findings enhance our understanding of cancer evolution and tumour suppression mechanisms with respect to body size. Furthermore, they may be usefully applied in future research for estimating cancer-related genes and functions.

## Supplementary Material

Table S1: List of mammals used in this study.

## Supplementary Material

Table S2: Correlation between NOGF and body size for all functional categories in which PICs were not considered.

## Supplementary Material

Table S3: Correlation between NOGF and body size for all functional categories in which PICs were considered.

## Supplementary Material

Figure S1: Phylogenetic tree of the 32 mammals used in this study.

## Data accessibility

The datasets supporting this article have been uploaded as part of the electronic supplementary material. The datasets are also deposited at Dryad (http://dx.doi.org/10.5061/dryad.8j0kf).
