# Supplementary material for: Importance of metabolic rate to the relationship between the number of genes in a functional category and body size in Peto's paradox for cancer
Source: R Soc Open Sci. 2016 Sep 7;3(9):160267. doi: 10.1098/rsos.160267 (PMC5043308; doi:10.1098/rsos.160267)
Supplement: Figure S1: Phylogenetic tree of the 32 mammals used in this study. [file rsos160267supp4.docx]

**Figure S1. Phylogenetic tree of the 32 mammals used in this study.**

The node labels correspond to the KEGG organism identifier. The tree presented in the Newick format is as follows:

(((((mmu:0.08104,rno:0.08248):0.04951,cge:0.12476):0.05344,ngi:0.11493):0.03966,(hgl:0.14409,(ocu:0.14687,(tup:0.13074,(((((((hsa:0.00253,ptr:0.00570):0.00045,ggo:0.00678):0.00389,pon:0.01198):0.00043,nle:0.01098):0.00529,mcc:0.02861):0.01593,cjc:0.04209):0.04991,(((((aml:0.00719,umr:0.00536):0.02221,cfa:0.03616):0.01115,(fca:0.00893,ptg:0.00972):0.03536):0.03039,((((((chx:0.01017,oas:0.00724):0.00583,phd:0.00768):0.01267,bta:0.02947):0.04670,(bacu:0.01987,lve:0.02461):0.02658):0.01069,ssc:0.07818):0.00211,cfr:0.07260):0.01699):0.00196,(myb:0.10819,(ecb:0.07922,pale:0.06770):0.00146):0.00192):0.02142):0.00257):0.00987):0.00324):0.02469):0.08808,(mdo:0.10233,shr:0.10678):0.13721).
